# Supplementary material for: Controlling the Switch from Neurogenesis to Pluripotency during Marmoset Monkey Somatic Cell Reprogramming with Self-Replicating mRNAs and Small Molecules
Source: Cells. 2020 Nov 5;9(11):2422. doi: 10.3390/cells9112422 (PMC7694496; doi:10.3390/cells9112422)
Supplement: Supplementary file 1 [file cells-09-02422-s001.zip › Supplementary_files/Figure S2.pdf]

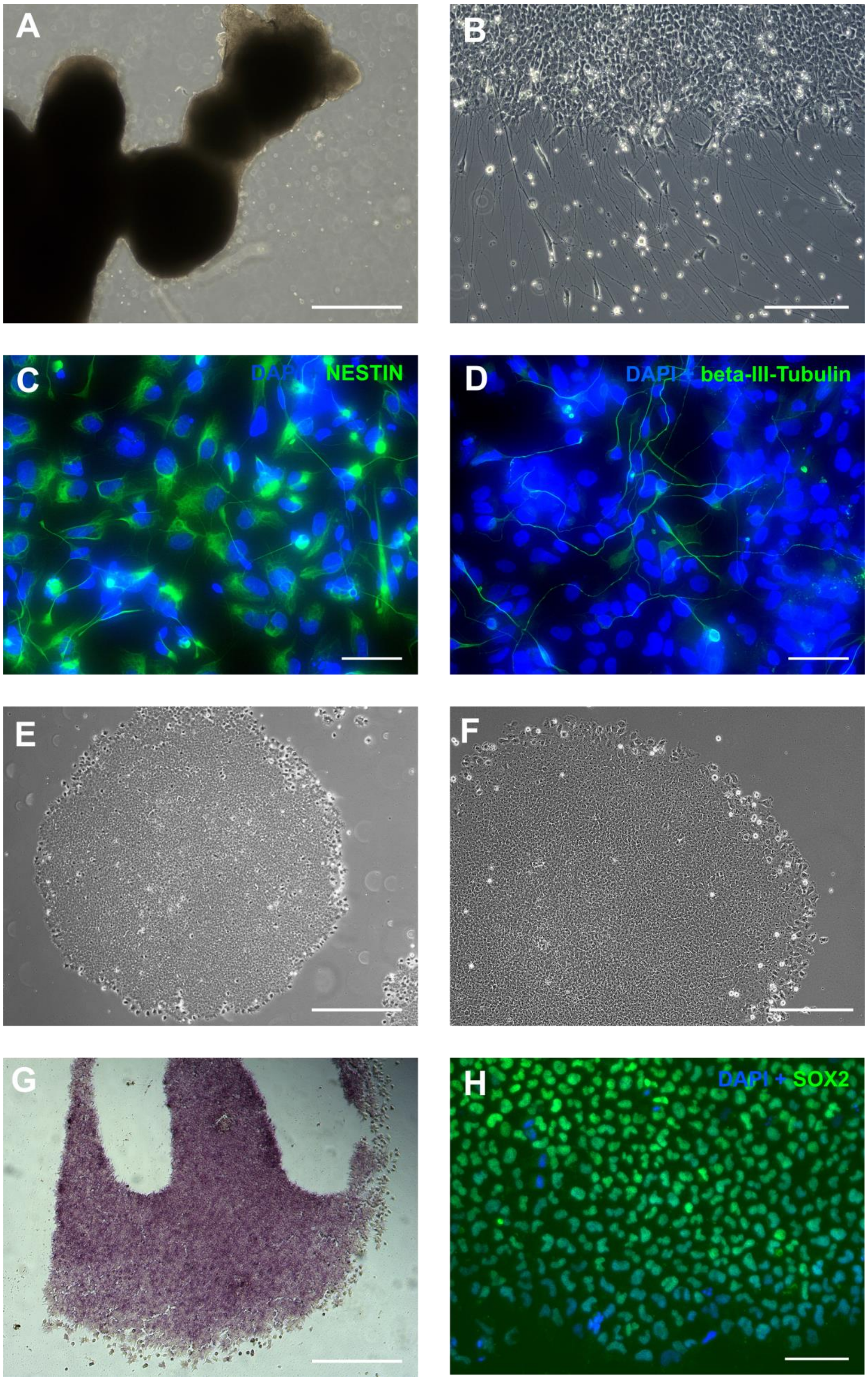


**Figure S2.** Embryoid body test and long-term culture of intermediate primary colony cells. **(A)** Suspension culture of primary colony fragments in Iskove's medium. **(B)** Outgrowths from the aggregates shown in (A) on Geltrex in Iskove's medium. **(C)** Immunofluorescence of outgrown cells stained with anti-NESTIN. **(D)** Immunofluorescence of outgrown cells stained with anti-β-III-Tubulin. **(E and F)** Putative neural progenitors derived from intermediate primary colony cells cultured long-term in iPS-Brew supplemented with 3 μM CHIR99021 and 10 μM SB431542. **(G)** Putative neural progenitor colony stained for alkaline phosphatase activity (parts of the colony have been scraped off with pipet tip and passed into new dish in order to continue the propagation). **(H)** Putative neural progenitor colony immunofluorescence with anti-SOX2 antibody. (Scale bars: A = 100 μm; B = 50 mm; C and D = 50 μm; E = 100 μm; F = 50 μm; G = 100 μm; H = 50 μm).
